# Editorial: Stem cell transplantation in autoimmune diseases (AID)

**DOI:** 10.3389/fimmu.2023.1150664

**Published:** 2023-03-01

**Authors:** Marc Schmalzing, Joerg Henes, Jacob M. van Laar, Keith M. Sullivan

**Affiliations:** ^1^ Rheumatology/Clinical Immunology, Department of Internal Medicine II, University of Würzburg, Wuerzburg, Germany; ^2^ Centre for Interdisciplinary Clinical Immunology, Rheumatology and Auto-inflammatory Diseases and Department of Internal Medicine II, University Hospital Tuebingen, Tuebingen, Germany; ^3^ Department of Rheumatology and Clinical Immunology, University Medical Center Utrecht, Utrecht University, Utrecht, Netherlands; ^4^ Department of Hematologic Malignancies and Cellular Therapy, Duke University Medical Center, Durham, NC, United States

**Keywords:** stem cell transplant (SCT), autoimmune disease, systemic sclerosis, mesenchymal stem cell, relapse

Around 25 years ago, studies in mouse models of autoimmune disease and case reports and case series describing successful outcomes of (mostly allogeneic) bone marrow transplantation in patients with hematological malignancies and concomitant autoimmune disease showed the potent effects of lymphoablative and myeloablative conditioning and stem cell rescue. As a result, autologous stem cell transplantation (aSCT) was introduced into the treatment armamentarium of autoimmune diseases, and has been increasingly used since. As our understanding of autoimmune diseases grew, many alternative treatment options became available and aSCT was shown to be less effective in several inflammatory rheumatic diseases like rheumatoid arthritis or juvenile idiopathic arthritis. However, aSCT still constitutes a cornerstone in the treatment of aggressive systemic sclerosis and other related connective tissue diseases due to the dismal prognosis and lack of efficacious alternatives. In systemic sclerosis, three randomized controlled trials have shown improved efficacy as to skin and lung involvement, quality of life and survival (1–3).

Due to frequent cardiopulmonary disease manifestations and exposure to hyperhydration, Antithymocyte globulin (ATG) and high dose cyclophosphamide during intensive immunoablative conditioning, treatment toxicity and risk of infections exceeded what was regarded as common in aSCT for hematological indications. Although successful efforts were made to reduce treatment related mortality – e.g. to roughly 6% in patients with systemic sclerosis - it still constitutes a relevant risk (4).

There are several questions remaining, requiring thorough research. These efforts are hampered by the rarity of appropriate disease subgroups, and the sheer effort and complexity of randomized aSCT studies in AID.

Several questions remain on the research agenda, such as:

- How can the success of mobilization regimes be increased?
- How can toxicity of condition regimens be reduced without losing efficacy?
- Is CD34 selection or ATG application necessary at all? Is dose reduction of ATG a way to reduce toxicity?
- How can the considerable risk of infections be reduced?
- How do we treat relapses after aSCT, or do we need maintenance therapy?
- Do we really achieve an immunological reset, and how long does it last?
- What are the mechanisms of the development of secondary autoimmune diseases after aSCT?
- Are there alternative (cellular) treatment options in autoimmune diseases, particularly with a better safety profile?

In this Research Topic, we collected valuable contributions to some of these questions.

A crucial aspect to increase benefit – risk ratio of aSCT in AID is the pre transplant selection of patients. Unfortunately, risk of progression of organ manifestations and future treatment response to aSCT remains hard to predict in the individual patient. Broens et al. present a review on the promising potential role of positron emission tomography to inform this evaluation.

There is little evidence on treatment of relapse or disease progression after aSCT for SSc. Relapse rates vary 10 and 40% depending on disease-related factors and treatment protocol (1, 2, 5). In an unicentric cohort presented by Gernert et al. nine of seventeen patients who received immunosuppressive treatment after aSCT were treated with rituximab with promising results. For more severe relapses, one might even consider a second aSCT. Haverkort et al. describe such a case with detailed assessment of treatment response.

As to alternative cellular treatment options for AID, CAR-T-cells have already been used in systemic lupus erythematosus with seemingly good benefit (6), and clinical studies on mesenchymal stem cells (MSC) dating back to the 90-ies held promise yet (7). Nevertheless, adequately powered controlled trials with MSC have not been published, and their mechanism of action remains unclear in parts. Li et al. and Xing et al. present their studies on the effect of MSCs and their exosomes respectively in animal models of Sjoegren’s Syndrome. Hopefully, preclinical studies of novel cellular therapies will move rapidly and successfully into the clinic.

Apparently, this Research Topic could not address all research agenda mentioned above. Therefore, the guest editors hope that it will stimulate further work in this field in the future.

## Author contributions

All authors listed have made a substantial, direct, and intellectual contribution to the work and approved it for publication.
